# A Fast Cluster Motif Finding Algorithm for ChIP-Seq Data Sets

**DOI:** 10.1155/2015/218068

**Published:** 2015-07-05

**Authors:** Yipu Zhang, Ping Wang

**Affiliations:** Department of Automation, School of Electronics and Control Engineering, Chang'An University, Xi'an 710064, China

## Abstract

New high-throughput technique ChIP-seq, coupling chromatin immunoprecipitation experiment with high-throughput sequencing technologies, has extended the identification of binding locations of a transcription factor to the genome-wide regions. However, the most existing motif discovery algorithms are time-consuming and limited to identify binding motifs in ChIP-seq data which normally has the significant characteristics of large scale data. In order to improve the efficiency, we propose a fast cluster motif finding algorithm, named as FCmotif, to identify the (*l*,  *d*) motifs in large scale ChIP-seq data set. It is inspired by the emerging substrings mining strategy to find the enriched substrings and then searching the neighborhood instances to construct PWM and cluster motifs in different length. FCmotif is not following the OOPS model constraint and can find long motifs. The effectiveness of proposed algorithm has been proved by experiments on the ChIP-seq data sets from mouse ES cells. The whole detection of the real binding motifs and processing of the full size data of several megabytes finished in a few minutes. The experimental results show that FCmotif has advantageous to deal with the (*l*,  *d*) motif finding in the ChIP-seq data; meanwhile it also demonstrates better performance than other current widely-used algorithms such as MEME, Weeder, ChIPMunk, and DREME.

## 1. Introduction

A Transcription Factor (TF) binds to the specific DNA sequences, which carries the information of the transcription and gene expressions. Hence locating the Transcription Factor Binding Sites (TFBSs) is crucial for uncovering the underlying relationship of regulating transcription and comprehending evolutionary mechanism of living organisms. The identification of TFBSs, or socalled motif discovery, is an issue of discovering short similar nucleotide segments with a common biological function. The planted (*l*, *d*) motif discovery is the famous version for motif discovery [1], which can be formulated as follows: given a set of *n*-length DNA sequences **S** = {*s*_*i*_∣*i* = 1,…, *t*} over the alphabet Σ = {A, C, G, T}, two nonnegative integers *l* and *d*  (*d* < *l* < *n*), where *l* is the length of a motif and *d* is the maximum number of mutations between the motif and a predicted binding site. The task is to find a *l*-length motif *m* occurring in most of the sequences including up to *d* mutations. *m* is called an (*l*, *d*) motif and each occurrence of *m* is called a motif instance. Various motif discovery algorithms have been developed to locate motifs in promoter sequences from coregulated or homologous genes based on either Consensus or Position Weight Matrix (PWM) [2].

In recent years, high-throughput technique ChIP-seq [3, 4], which couples chromatin immunoprecipitation experiment [5] with high-throughput sequencing technologies, has extended the identification of binding locations of a given TF to that of the genome-wide regions. The genome-wide ChIP experiment generally produces thousands of sequences of a few hundred bps (ChIP-seq peaks), which provides data set of one or two magnitudes larger than a typical motif discovery data set and sequences with a high resolution. The novel ChIP technique ChIP-exo can locate binding sites at a higher resolution, but its binding regions identified by ChIP-seq or ChIP-exo experiments may be dozens of bps away from the true binding sites [6]. Computational motif discovery methods are still needed to identify the binding locations of a TF in ChIP-seq or ChIP-exo data sets [7] in the high accuracy.

In order to detect motifs in large-scale ChIP-seq data, some traditional motifs discovery algorithms have been proposed in their ChIP-tailored versions, such as MDscan [8] and MEME-ChIP [9]. These algorithms normally find motifs by using a limited part of the sequences, while ignoring the remaining unselected sequences. That decreases the chance of discovering motifs related to infrequent cofactors. Meanwhile, PWM-based methods also have been developed. For instance, STEME [10] applies suffix trees to accelerate EM steps. This strategy acts well in case of finding short motifs. However, it executes much slower when the width of motif increases in the large data set. HMS [11] is an improved version of Gibbs that combines sampling algorithms with greedy search steps. ChIPMunk [12] introduces EM algorithms with a greedy approach and applies a more complex statistic model. These algorithms aim to optimize a PWM of ChIP-enriched region. They still have an unsolved problems of local optimum and the iteratively training also costs too much. Additionally, consensus-based algorithms are designed based on word-enumeration methods, such as RAST [13] and CisFinder [14], which can process whole ChIP-seq data set by two contrastive data sets. Both RAST and CisFinder are limited to find short motifs and may miss the useful information contained in the sequences.

To overcome these shortcomings, in this paper, we propose a fast cluster motif finding algorithm, named FCmotif, to solve the (*l*, *d*) motif identification problem in large scale ChIP data set. FCmotif utilizes the emerging substrings mining strategy to find the enriched substrings at first and makes each emerging substring as a reference core to construct PWM. Then our algorithm uses the constructed PWMs to cluster the motifs in different length, and we consider intramotif dependency in statistics model to calculate information content (IC) and false discovery rate (FDR) to optimize the outputs. FCmotif achieves to deal with the whole data set that does not limit to the OOPS (one occurrence of the motif instance per sequence) constraint. The experimental results show that FCmotif is advantageous to deal with the (*l*, *d*) motif finding in the ChIP-seq data, and it also demonstrates better performance than other current widely-used algorithms such as MEME, Weeder, ChIPMunk, and DREME.

## 2. Materials and Methods

We know that the characteristic of a ChIP-seq data set is a large scale set of relative shorter sequences. That is, the amount and quality of ChIP-seq data have been dramatically increased. Each sequence of ChIP-seq data set contains less “the background information,” and several instances of the motifs could be expected to exist in thousands of sequences. From this point of view, our main objective is to handle the whole data set and distinguish the motif instance from the relative “cleaner” background sequence.

### 2.1. Motif Representation

Generally, a motif can be represented by a PWM Θ, of which each column stores the occurring frequency of the four types of nucleotides (Σ = {A, C, G, T}). Let Θ = (*θ*_1_,…, *θ*_*l*_), where *θ*_*i*_ represents the probability of nucleotide preference at the *i*th position of the motif, and let *θ*_0_ be the probability of nucleotide observing at the nonmotif positions in the sequences. For each substring of *l* length (we also call *l*-mer) *s* = *s*_1_,…, *s*_*l*_, the log-likelihood of letter *s*_*i*_ at position *i* is given by

(1) $$\begin{array}{c} p\left(\;s_{i}\;\right)=\log\frac{\theta_{ik}}{\theta_{0k}}, \end{array}$$

where *θ*_*ik*_ is the probability of observing letter *s*_*i*_ = *k* at position *i* and *θ*_0*k*_ is the background probability of letter *k*. This classical product-multinomial model proposed by Liu et al. [15] has been widely used in* de novo* statistic algorithms such as EM and Gibbs algorithm. It assumed that the positions within the motif are independent of each other [16]. However, recent researches imply that the commonly used product-multinomial model may be too simplistic in identifying the binding motifs, while some positions of TF binding motif exert an interdependent effect on binding affinities of TFs [17–19].

To provide a better fit model to increase the quality of motifs identified by ChIP-Seq, a more sophisticated model that involves the intramotif dependency should be considered. Here, “intramotif dependency” means that the frequency of nucleotide combinations spanning several positions deviates from the expected frequency under the independent motif distribution [11]. For instance, if the frequency of two nucleotides, “GT,” in a pair of positions is much higher or lower than the product of frequency of “G” in the first position and frequency of “T” in the second position, we infer that these two positions are dependent. Here, we implement a 16-component dependent multinomial model to scan each pair of positions within the motif to determine the intramotif dependency. Let Φ_*i*,*i*+1_ represent the probability of observing nucleotide pair at *i*th and (*i* + 1)th position of the motif. For each pair of positions, there are *l*-1 dependent multinomial distributions to be estimated. The log-likelihood of letters *s*_*i*_, *s*_*i*+1_ at position *i* and *i* + 1 is

(2) $$\begin{array}{c} p\left(\;s_{i},s_{i+1}\;\right)=\log\frac{\Phi_{i,i+1}\left(s_{i},s_{i+1}\right)}{\Phi_{0}\left(s_{i},s_{i+1}\right)}, \end{array}$$

where Φ_0_ represents the background probability of the nucleotide pair. The Log-Likelihood Ratio (LLR) of *l*-mer *s* is then

(3) LLRs=log⁡Us·Vsp0s,

(4) Us=∏i=1l ∏k∈Σθik,

(5) Vs=∏i=1l−1 ∏k1,k2∈ΣΦi,i+1k1,k2.

Here, formula (4) represents the joint probability of the independent nucleotides in motif, and formula (5) represents the joint probability of the nucleotide pair in motif. Formula (3) is the LLR of *s* under the corresponding background distribution *p*_0_. For the background (nonmotif) regions, we employ a high-order Markov model to obtain the weak dependency in background DNA sequences. Compared with the uniform distribution or random distribution background, the high-order Markov model can improve the sensitivity and specificity of identifying motifs. In this study, we use a third-order Markov model to characterize the background sequence. As an example, the probability of an *l*-mer *s* (*s*_*i*_, *s*_*i*+1_,…, *s*_*i*+*l*−1_) in the background under a third-order Markov model can be represented by

(6) p0spsipsi+1 ∣ sipsi+2 ∣ si,si+1⋯psi+l−1 ∣ si+l−2,si+l−3,si+l−4.

Thereby, the Information Content of motif can be represented as

(7) $$\begin{array}{c} \text{IC}=\underset{s\in \Sigma^{l}}{\sum }p\left(\;s\;|\;\Theta \;\right)\log\left(\;\frac{p\left(s\;|\;\Theta \right)}{p_{0}\left(s\;|\;\theta_{0}\right)}\;\right). \end{array}$$

### 2.2. Emerging Substrings Mining

For the large-scale data set, calculating the likelihood score of each substring costs too much, which makes probabilistic training methods unpractical. Pattern-driven strategy can use shorter time to count the substrings that have higher occurrence frequencies. Since each instance differs from motif at most *d* positions, we expect to find some instances occurring multiple times in thousands of sequences and reduce the disturbance of random overrepresented substrings. With the above considerations, we utilize both a test set and a control set of DNA sequences to search the possible motif instances. Generally, the test set consists of the sequences with motifs, while the control set contains the background sequences. The interested substrings are the ones that present in the test set and absent in the control set, and we call such substrings emerging substrings. The task converts to solve emerging substrings mining problem [20] and then identifies motif instances from the emerging substrings. The emerging substrings mining problem is defined as follows.

Given a test set **S**_*t*_ and a control set **S**_*c*_ of sequences over the alphabet Σ = {A, C, G, T}, frequency threshold *λ*_*f*_  (1/|**S**_*t*_| ≤ *λ*_*f*_ ≤ 1), and growth rate threshold *λ*_*g*_  (*λ*_*g*_ > 1), the task is to find all substrings *u*  (*l*_min_ ≤ *u* ≤ *l*_max_) satisfying the conditions *f*(*u*, **S**_*t*_) ≥ *λ*_*f*_ and *g*(*u*, **S**_*t*_, **S**_*c*_) ≥ *λ*_*g*_ at the meantime. Such substrings are called emerging substrings. Here, *f*(*u*, **S**) represents the frequency of substring *u* occurring in set **S**, and *g*(*u*, **S**_*t*_, **S**_*c*_) = *f*(*u*, **S**_*t*_)/*f*(*u*, **S**_*c*_), that is, the growth rate of substring *u* from set **S**_*t*_ to set **S**_*c*_. Large value *g*(*u*, **S**_*t*_, **S**_*c*_) means that substring *u* is highly discriminative for two input data sets.

With the above material, our algorithm can be summarized as the following main procedures. First, we compare the substrings in both test set and control to obtain the emerging substrings. Second, calculate measure score of the emerging substrings to find the true motif instances. Nevertheless, there are still some key problems needed to be solved: (i) As the exact motif length is unknown, we need to select a range of emerging substring length to find motif. (ii) The interested emerging substrings contain true motifs, the instances of both mutation and random disturbance, how to reduce the influence of the unreal instances. (iii) We need to choose one model from OOPS, ZOOPS (zero- or one-motif occurrences per sequence), and TCM (two-component mixture) to find motif instances in each sequences. Therefore, our algorithm is designed in detail to further process the emerging substrings and handle these problems.

### 2.3. FCmotif Algorithm

**Step 1 (searching emerging substrings).**

An essential assumption is that the evidence for binding motif is large in test set and small in the control set. To streamline the predicting sites algorithm and handle the ChIP-Seq data, our algorithm utilizes pattern-driven word enumeration strategy to search the emerging substrings. Assume motif length is *l*; we first count the amount of all possible 4^*l*^ *l*-mers in both test set and control set; then we select the rich ones. The threshold frequency *λ*_*f*_ and growth rate *λ*_*g*_ are two important parameters employed in this step.

As previous studies [21, 22], we knew the probability of the occurrence of a random mutated instance *m*′ of a reference motif *m* at random *i* positions is

(8) $$\begin{array}{c} P_{d}\left(\;i\;\right)=\left(\begin{array}{c} d\\ i \end{array}\right)p_{\text{con}}^{i}{\left(\;1-p\;\right)}^{d-i},\;0\leq i\leq d, \end{array}$$

where *p*_con_ is the mutating probability, and it can also represent the conservation of motif. We set *p*_con_ as 0.2, 0.5, and 0.8 to represent high conservation, intermediate conservation, and low conservation, respectively.

Then, according to the definition of (*l*, *d*) motif, the probability of a random (*l*, *d*) instance *m*′ of motif *m* occurring in a sequence can be calculated by

(9) $$\begin{array}{c} P_{\text{occ}}=\overset{d}{\underset{i=0}{\sum }}P_{d}\left(\;i\;\right)\frac{1}{\left(\begin{array}{c} l\\ i \end{array}\right)\times 3^{i}}. \end{array}$$

Moreover, for the different models, each sequence contains different amount of motif instances, so the value of *λ*_*f*_ can be set by different models and *P*_occ_. We set *λ*_*f*_ = 0.8*P*_occ_ when model is OOPS, *λ*_*f*_ = 0.6*P*_occ_ when model is ZOOPS, and *λ*_*f*_ = 1.2*P*_occ_ for TCM. Meanwhile, the default value of *λ*_*g*_ that we set is 2.  Table 1 shows an example of searching the emerging substrings of length 6 in 600 sequences for ZOOPS model; (1, *d*) = (6,1) and *p*_con_ = 0.8. From the example, we can find that the emerging substring “CAGCGA” satisfies both *f*(*u*, **S**_*t*_) > *λ*_*f*_ and *g*(*u*, **S**_*t*_, **S**_*c*_) > *λ*_*g*_. However, only the emerging substring cannot indicate motif; it may miss the mutated instances especially for larger value of *l* and *d*.

**Step 2 (constructing the corresponding PWM).**

The emerging substrings can represent a part of the enriched (overrepresented) motifs. However, it still contains the fake instances made up by the background sequences and cannot reflect the true mutated (*l*, *d*) motif instances. It is necessary to measure the statistics scores of all possible instances and find the (*l*, *d*) instances among the emerging substring. The PWM indicates the distributions of each character at each position of the motif, and it is the core of measuring the statistical significance, so we construct the corresponding PWM of each emerging substring by the (*l*, *d*) mutating.

Assume each emerging substring is a reference motif; the motif instances should exist in the mutated *l*-mers at most *d* positions from the reference motif (called the “neighborhood” instances), which have a larger amount in the test set than that in the control set. When we find out the mutated instances from the reference motif, we can use them to construct the core PWM and measure the statistical scores. In this way, see each emerging substring as a reference; we first search its “neighborhood” instances and evaluate the *z*-score of each one. *z*-score is a statistical measurement of a score's relationship to the mean in a group of scores which is estimated based on the hypergeometric probability distribution [14]:

(10) $$\begin{array}{c} z\left(\;u\;\right)=\frac{q_{1}-q_{2}}{\sqrt{q\left(1-q\right)\left(N_{1}+N_{2}\right)/\left(N_{1}N_{2}\right)}}, \end{array}$$

where *q*_1_ = *C*_1_/*N*_1_, *q*_2_ = *C*_2_/*N*_2_, and *q* = (*C*_1_ + *C*_2_)/(*N*_1_ + *N*_2_). *C*_1_ and *C*_2_ represent the number of occurrences of *l*-mer *u* in **S**_*t*_ and **S**_*c*_, while *N*_1_ and *N*_2_ are the total number of *l*-mers in **S**_*t*_ and **S**_*c*_, respectively.

For convenience of description, here we give an example to explain the searching process. Consider a specific reference *l*-mer “ACCACGTG,” which has 119 matches in the test set and 46 matches (9 matches after adjusting test set and control set with the same size) in the control set. As the previous study [21], for the length *l* = 8, we use (*l*, *d*) = (8,1) to search the “neighborhood” instances. Therefore, we find the *l*-mers that have mutated to the other three characters at one position from the reference *l*-mer. Note that using (8, 1) model, we only need to search 24 *l*-mers to find the “neighborhood” ones but not the whole searching space of 4^*l*^  (65536, *l* = 8) *l*-mers.  Table 2 shows each neighborhood instance of the reference *l*-mer “ACCACGTG,” *z*-score, and the number of occurrences in the test and the control sets.

Use each emerging substring as a reference center and incorporate its neighborhood instances; two Position Count Matrices (PCMs) of size 4 × *l*, *M*_1_ and *M*_2_ can be formed. *M*_1_ is composed of the qualified neighborhood instances in **S**_*t*_, and *M*_2_ is similarly composed of the qualified neighborhood instances in **S**_*c*_ adjusted for length (rescaled by *N*_1_/*N*_2_). Here, the qualified neighborhood instances refer to the instances with *z* > 1.643 or the instances with the maximum positive *z*-score if there is no instances with *z* > 1.643 [23]. While the PCMs *M*_1_ and *M*_2_ are constructed by adding up the counts of “A, C, G, T” at each position of the qualified neighborhood instances.

**Figure 1:**
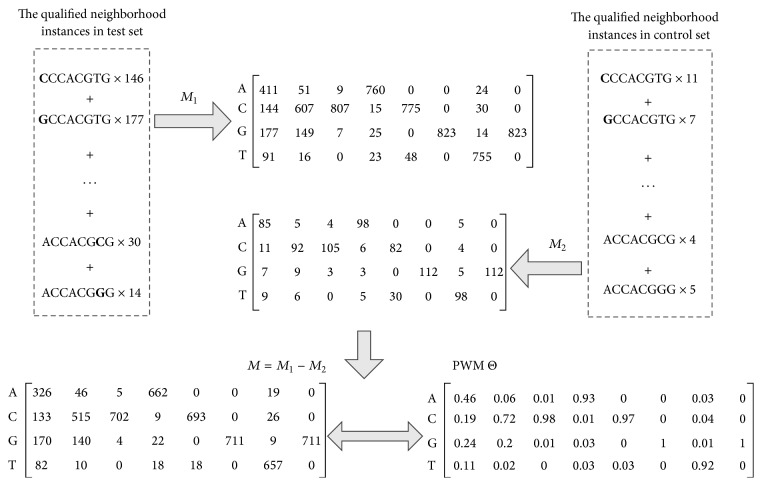
An example of constructing the PWM of the corresponding substrings.

It is worthy to note that the test set is a mix of motif instances and random disturbances of background, and the control set is full of background noise. Hence, we can use the background distribution found in the control set to recorrect the contribution and estimate the PWM in the test set. That is, *M*_2_ can be regarded as the expected count matrix constructed from false positive motifs in **S**_*t*_. In this way, let the PCM *M* = max⁡(*M*_1_ − *M*_2_, 0); we can get PWM Θ of the reference emerging substring by normalizing each row into probability distribution. In order to avoid zero frequency, 5% pseudo-counts to each position are added. As we also concerned the intramotif distribution in the probabilistic model, Φ can be estimated in the same way.  Figure 1 shows an example of constructing PWM of the corresponding emerging substring in Table 2.

**Step 3 (clustering longer motifs).**

See each emerging substring as a seed; its PWM can be obtained by the steps above, while the corresponding motif with high IC score can also be computed. However, the PWMs may represent many similar motifs with a few letters varying as previous studies [24, 25]. In order to eliminate redundant motif information and expand the short motif to form longer motif, we cluster the similar motifs and combine the motifs having the long common-overlap segments by utilizing a metric of computing the Euclidean distance between two *l*-mers as described below:

(11) $$\begin{array}{c} D\left(\;a,b\;\right)=\frac{1}{\sqrt{2}l}\overset{l}{\underset{i=1}{\sum }}\sqrt{\underset{k\in \Sigma }{\sum }{\left(a_{ik}-b_{ik}\right)}^{2}}, \end{array}$$

where *l* is motif length and *a*_*ik*_ and *b*_*ik*_ are the estimate probabilities of observing letter *k* at position *i* of *l*-mers *a* and *b*, respectively. Since the length of predicted motifs may be different, we actually use the minimum distance between motifs among all possible overlaps of motifs *a* and *b* induced by shifts that the minimum overlap is 7 bases or two bases fewer when the motifs are even shorter. Hence, we use the Harbison similarity score [26]:

(12) $$\begin{array}{c} \mathrm{sim}\left(\;a,b\;\right)=\underset{a^{\prime },b^{\prime }}{\max}\left[\;1-D\left(\;a^{\prime },b^{\prime }\;\right)\;\right], \end{array}$$

where *a*′ and *b*′ correspond to all possible overlaps of *l*-mers *a* and *b*. In this way, two *l*-mers *a* and *b* are considered similar if the PWMs of *a* and *b* have the Harbison similarity score ≥ 0.75. In practice, as the motif length is unknown, we use *l*  (*l*_min_ ≤ *l* ≤ *l*_max_) in a proper range and cluster the PWMs of different length which satisfy the Harbison similarity score constraint. So in this step, a longer motif can be obtained by the corresponding PWM that is combined by clustering the PWMs of different *l*.

**Step 4 (output).**

With the combined PWMs, we employ two measures to optimize the motifs; first we compute IC and then utilize the False Discovery Rate (FDR) to control the final outputs. The False Discovery Rate as a function of the threshold *μ* can be intuitively defined as

(13) $$\begin{array}{c} \text{FDR}\left(\;\mu \;\right)=\frac{I_{2}\cdot N_{1}/N_{2}}{I_{1}}, \end{array}$$

where *I*_1_ = ∑_*s*∈**S**_*t*__ *I*(LLR(*s*) > *μ*) is the number of *l*-mers found in **S**_*t*_ and  *I*_2_ = ∑_*s*∈**S**_*c*__ *I*(LLR(*s*) > *μ*) is the number of *l*-mers found in **S**_*c*_. LLR(*s*) can be calculated by formula (3). Here, we define *μ* as an integer satisfying 0 ≤ *μ* ≤ max[LLR_*s*∈**S**_*t*__(*s*)] which leads to FDR(*μ*) < 0.2 (FDR value changes with different data sets). Once *μ* is determined, the *l*-mers in **S**_*t*_ with LLR(*s*) > *μ* are the predicted motif instances. In practice, we finally generate at least 50 top IC score motifs by formula (7) satisfying the FDR constraint.

Main algorithm of FCmotif is shown in Algorithm 1.

In Step 1, lines (6) to (9), we find the emerging substrings enriched in the test set; then lines (10) to (15) are the step to construct the PWM and the intramotif distribution for each emerging substring. Lines (16) to (19) are the step to cluster PWM with the similar Harbison similarity score. Lines (20) to (24) are the last step to compute IC and FDR and finally output the result.

## 3. Results and Discussion

We use the ChIP-seq data sets of 12 TFs profiled in mouse ES cells [27] to test the validity of our algorithm. These 12 data sets are key to the maintenance of pluripotency, in which Nanog, Oct4, Sox2, Esrrb, and Zfx are regulators of self-renewal; Klf4, cMyc, and mMyc are the crucial reprogramming factors [28, 29]; Tcfcp2l1 is preferentially upregulated in ES cells [30]; Smad1 and STAT3 have the significant meaning to the signalling pathways, and CTCF is a key component for transcriptional insulation [31]. For the test set, we extract 200 bps sequence segments centered at a peak of TF location. For the control set, we extract 500 bps sequence segments starting from nucleotide positions 400 bps away from both ends of 200 bps positive sequence segments. The total sizes range from 1 Mb to 50 Mb.  Table 3 is the statistics information about the 12 mES ChIP-seq data sets.

Our algorithm runs by using the following parameters: word enumeration analysis is performed with the length *l* ranging from 6 to 12. *λ*_*f*_ = 0.8*P*_occ_ when model is OOPS, *λ*_*f*_ = 0.6*P*_occ_ when model is ZOOPS, and *λ*_*f*_ = 1.2*P*_occ_ for TCM. *λ*_*g*_ = 2 while *P*_occ_ = 0.2, 0.5, and 0.8 to represent high conservation, intermediate conservation, and low conservation, respectively. The (*l*, *d*) settings we used include (6,1), (7,1), (8,1), (9,2), (10,2), (11,2), and (12,3). The default value of threshold *z* for selecting qualified neighbourhood instances is 1.643, and the FDR constraint is 0.2. FCmotif is implemented in Matlab under the experiment environment: 2.67 Hz CPU and 4 G memory.

### 3.1. Results on 12 TF Binding Sites in mES Cells

**Figure 2:**
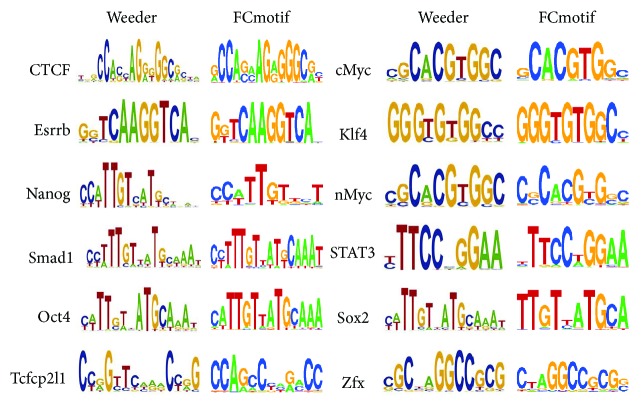
The logos of primary motifs predicted by Weeder and FCmotif.

**Figure 3:**
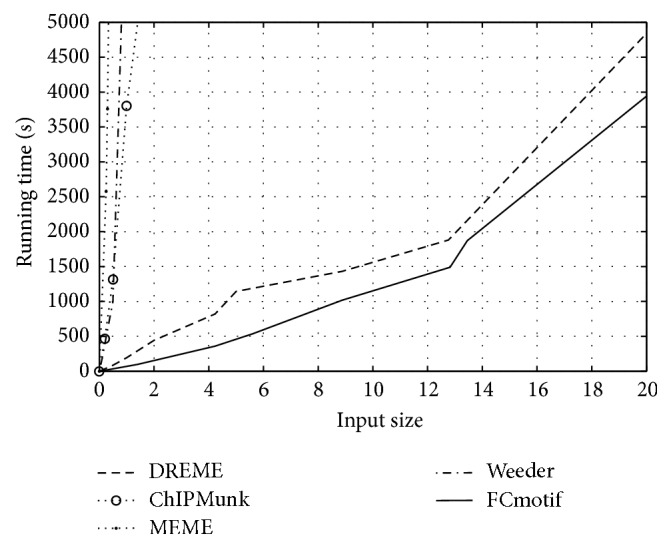
The running time for FCmotif, DREME, ChIPMunk, MEME, and Weeder on the full-size mESCChIP-seq data sets.

To evaluate the performance of our algorithm, we compare the primary motifs of 12 TFs in mES ChIP-seq data sets discovered by our algorithm with the motifs found by Chen et al. with Weeder. The motif comparison is performed by comparing matrices [32], which supports various scoring metrics and shows the results as the logo of aligned words, in order to grasp the similarities between a predicted motif and the known motifs.  Figure 2 shows all 12 motifs identified by FCmotif and motifs found by Chen et al., indicating that the quality of results is comparable. Moreover, we also compare the running time on our algorithm with that of the popular motif discovery algorithms, MEME [33], Weeder [34], ChIPMunk [12], and DREME [35].  Figure 3 shows the running time of above algorithms of 12 mES ChIP-seq data sets. Note that both MEME and Weeder are too slow to deal with ChIP-seq data sets containing thousands of sequences and often fail after running for many days. For this reason, the results in Figure 3 for MEME and Weeder are obtained on the reduced-size data sets. ChIPMunk is an iterative algorithm which can process up to tens of thousands of sequences but with enormous computation at the same time. DREME can predict more accurate motifs than the traditional motif discovery algorithm but was restricted to 500 top-scoring peaks; it cannot analyze the full size data sets. For our algorithm, we found that the computing time scales up efficiently with sequences size and our algorithm outperforms all the compared motif discovery algorithms. Data of several megabytes can be processed in a few minutes. In addition, it is worth to point out that the word length *l* is another factor to influence the computational efficiency. Because the number of possible *l*-mers and the number of neighborhood instances are increasing dramatically with *l* increasing. For example, for Sox2 data set, running time when *l* = 8 is 536 s but 620 s when *l* = 10. CisFinder [14] is another algorithm that uses the idea of word enumeration and compares the word enrichment of two input sets; it works extremely fast. However, CisFinder outputs the motifs using *p*-value as a measure, which cannot reflect the significance of the motif but only a single word matching the motif [35].

**Figure 4:**
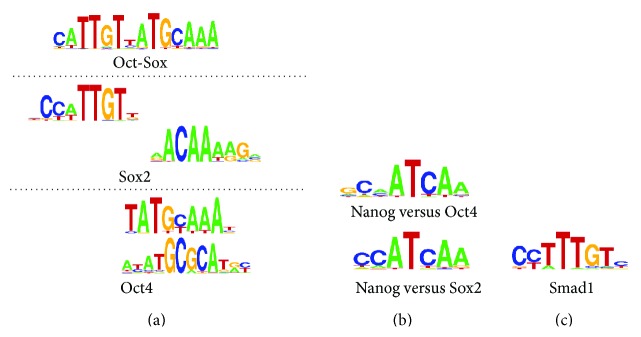
Multiple motifs discovered by FCmotif. (a) The Oct-Sox composite motif, alternative motifs of Sox2, and alternative motifs of Oct4 found by FCmotif. (b) The discriminative motifs found by FCmotif using Nanog data set as the test set, the Oct4 data set, or the Sox2 data set as the control set. (c) The extra motif found by FCmotif in Smad1 data set.

Although the analysis from 50 to 200 top-scoring binding sites is sufficient to extract the primary motif, yet this data size usually used by Weeder or MEME is not enough to examine alternative motifs. For instance, in Sox2 and Oct4 data sets, Chen et al. report only a single motif with Weeder, respectively. In contrast, FCmotif can find multiple motifs for each TF using the same data sets. As shown in Figure 4(a), our algorithm predict not only the Oct-Sox composite motif bound by Sox2 and Oct4 complex [27] but also the characteristic motifs Sox2 (CCATTGTT) and Oct4 (TATGCAAAT). Meanwhile, some predicted motifs, with no similarity with the prevalent consensus of the data set, often have a high significance and may reveal alternative consensus. Such as the motif (ATATGCGCATGC) in the Oct4 data set, it corresponds to an alternative Oct4 motif reported in other studies [13, 35].

Nanog and Smad1 data sets also have nearly the same binding regions like Sox2 and Oct4 data sets as discussed by Chen et al. As shown in Figure 2, the significant motif found in Nanog is Oct-Sox, which means these similarity binding regions may cause Nanog motif to bind indirectly via one or both of Sox2 and Oct4 TFs and raise the difficulty to identify motifs of the TFs. Therefore, the approach to find the enriched motifs in Nanog data set relative to Sox2 or Oct4 data sets is needed. Here, we use Nanog ChIP-seq data set as the test input and either of Sox2 and Oct4 data sets as the control input. From the results shown in Figure 4(b), the significant predicted motifs of these two compared data sets are similar, and both of them are also similar to the previously reported motifs “CCATCA” by [23, 35] as an alternative Nanog motif.

In addition, for Smad1 data set, our algorithm not only discovers a motif “AACAAAGC” matching the published Smad1 motif “AAACAAAG” but also finds other motifs, like “CCTTTGTC”, which matches a Sox2 motif (Figure 4(c)). And these discovered motifs demonstrate the frequent cobinding relationship of Smad1 and Sox2 TFs.

In contrast with the traditional analysis of transcriptional regulation that motifs commonly bind to a DNA-binding TF, genome-wide locations for a specific TF usually do not carry the primary or alternative binding motif but the binding motifs for other TFs. To explore this issue, we employ the ChIP-seq approach to characterize these TFs that bind to DNA indirectly through binding to a cofactor. We use a histone acetyltransferase generally found at enhancer regions [27], P300, to reveal the interaction of cofactors and DNA-binding TFs, and hope to infer the potential tissues of transcription regulation. From the results, we found that P300 does not only associate with the notable Oct4, Sox2, Nanog, and Smad1 TFs but also cooccurs with Oct-Sox complex and other abundant TFs including a TEF motif “AGGATTGCT” and the core of AP4-L motif “CAGCAGG.” In addition, there are still several motifs found by our algorithm in relative low probabilities, such as Esrrb motif “GAgGGTgA,” Klf4 motif “GGTGTGGg,” and Tcfcp2l1 motif “CCAGTTgcA.”

The results of experiment show that our algorithm makes a good trade-off between accuracy and efficiency. It shows better performance than the other compared algorithms. Data of several megabytes can be handled in a few minutes; when data is up to 50 Mb, it can be handled in several hours. We can see in the word enumeration that FCmotif counts all the *l*-mers in both test and control sets. Suppose the both sets have the same size: the sequence length *n* and the number of the sequences *t*, and the motif length is *l*, so the computational complexity of counting all the *l*-mers is *O*(*ntl*) which is completely acceptable. The step of searching emerging substrings dramatically reduce the number of potential motif instances; generally, the order of magnitude of emerging substrings is *O*(10^2^). Moreover, note that the range of *l* is from 6 to 12 bps, the (*l*, *d*) values include (6,1), (7,1), (8,1), (9,2), (10,2), (11,2), and (12,3), and the number of the neighborhood instances *E* is

(14) $$\begin{array}{c} E=C_{l}^{1}3+C_{l}^{2}3^{2}+\cdots +C_{l}^{d}3^{d}. \end{array}$$

So the maximum *E* is 6570 for (*l*, *d*) = (12,3), which means our algorithm does not need to search thousands of possible instances to form PWM and can limit the number of enriched *l*-mers in several dozens. For a few megabytes data, our algorithm can search out a fixed length motif in a few minutes with the amount of computation in *O*(10^8^) or *O*(10^9^) (for a large *l*), which is more faster than that of the probability training methods. In addition, recent studies indicate that many regulatory regions are located in transposable elements which are commonly not conserved [36].

## 4. Conclusions

In this paper, we propose a fast cluster motif finding algorithm, named FCmotif, to solve the (*l*, *d*) motif identification problem in large scale ChIP data set. FCmotif overcomes drawbacks of traditional algorithms which are time-consuming and cannot handle the full size data; it guarantees to find all potential (*l*, *d*) motif instances. FCmotif utilizes a word enumeration strategy and searches the neighborhood instances to form the PWM of enriched substrings. It breaks up constrain of the OOPS model and can find long motifs by clustering the PWM in different lengths. The experiments of the ChIP-seq data sets from mouse ES cells confirm that FCmotif can find not only the primary motif but also the exceptional motifs, which uses less time compared to popular motif discovery algorithms. Meanwhile, the potential cobinding relationship can be also detected by our algorithm. It is worth noting that our algorithm is easy to parallel because the calculation of motif of each length is independent.

In summary, it can be seen that FCmotif is a competitive algorithm to deal with the (*l*, *d*) motif finding in the ChIP-seq data. The functions of some motifs found by our algorithm are still unknown. The functions of some motifs found by our algorithm are still unknown, the further experimental validation is needed to prove that these motifs are indeed functional. The analysis of motifs in these complex transcriptional regions is the key issue for the future study.

## Figures and Tables

**Algorithm 1:**
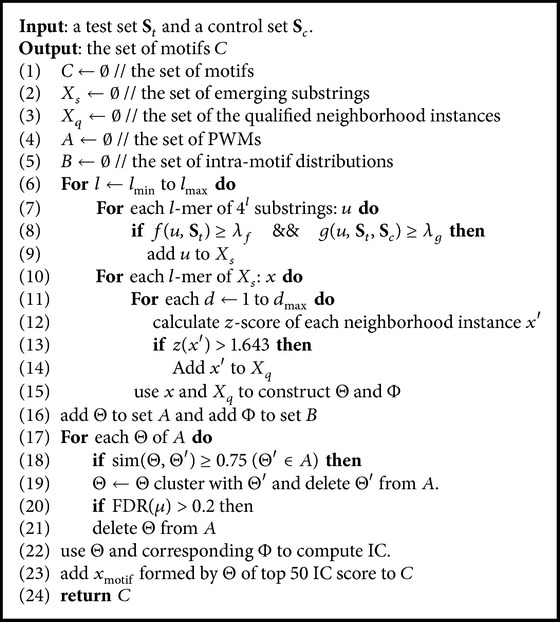


**Table 1:** An example of searching the emerging substrings of all possible *l*-mers.

*l*-mer	Number in test set	Number in control set	*f*(*u*, **S**_*t*_)	*g*(*u*, **S**_*t*_, **S**_*c*_)	*λ*_*f*_ = 0.02667 *λ*_*g*_ = 2
AACTGC	5	16	0.0083	0.3125	N
AAGTGG	8	6	0.0133	1.3333	N
CAGCGA	19	3	0.0317	6.3333	Y
TGACTT	15	7	0.025	2.1429	N
GCTTCA	2	5	0.0033	0.4	N
⋮	⋮	⋮	⋮	⋮	⋮

**Table 2:** An example of searching the neighbourhood instances of a reference *l*-mer.

Reference *l*-mer: ACCACGTG (*l*, *d*) = (8, 1) *z* = 19.36 *C*_1_ = 119 *C*_2_ = 9									
Position	Instance	*z*	*C*_1_	*C*_2_	Position	Instance	*z*	*C*_1_	*C*_2_
*i* = 1	***C***CCACGTG	**21.78**	146	11	*i* = 2	A***A***CACGTG	**11.83**	51	5
	***G***CCACGTG	**26.14**	177	7		A***G***CACGTG	**22.66**	149	9
	***T***CCACGTG	**15.93**	91	9		A***T***CACGTG	**3.28**	16	6

*i* = 3	AC***A***ACGTG	**2.38**	9	4	*i* = 4	ACC***C***CGTG	**2.88**	15	6
	AC***G***ACGTG	**1.82**	7	3		ACC***G***CGTG	**8.23**	25	3
	AC***T***ACGTG	1.31	5	3		ACC***T***CGTG	**6.16**	23	5

*i* = 5	ACCA***A***GTG	−0.73	7	9	*i* = 6	ACCAC***A***TG	0.14	27	26
	ACCA***G***GTG	−0.12	14	14		ACCAC***C***TG	−0.30	17	18
	ACCA***T***GTG	**2.88**	48	30		ACCAC***T***TG	−2.08	2	8

*i* = 7	ACCACG***A***G	**5.92**	24	5	*i* = 8	ACCACGT***A***	−0.07	3	3
	ACCACG***C***G	**8.63**	30	4		ACCACGT***C***	1.03	7	5
	ACCACG***G***G	**3.53**	14	5		ACCACGT***T***	−0.05	4	4

**Table 3:** The information of 12 mES ChIP-seq data sets.

TF	Peaks	Total size (Mb)	Running time (sec)
CTCF	39601	48.98	20570 s
cMYC	3422	4.23	360 s
Esrrb	21644	26.83	6101 s
Klf4	10872	13.45	1871 s
Nanog	10342	12.82	1489 s
nMyc	7181	8.88	1018 s
Oct4	3761	4.65	426 s
STAT3	2546	3.14	295 s
Smad1	1126	1.40	98 s
Sox2	4526	5.60	536 s
Tcfcp2l1	26907	33.59	11075 s
Zfx	10336	12.76	1310 s
